# Supplementary material for: Novel NPR2 Gene Mutations Affect Chondrocytes Function via ER Stress in Short Stature
Source: Cells. 2022 Apr 8;11(8):1265. doi: 10.3390/cells11081265 (PMC9024524; doi:10.3390/cells11081265)
Supplement: Supplementary file 1 [file cells-11-01265-s001.zip › cells-1631981-supplementary.pdf]

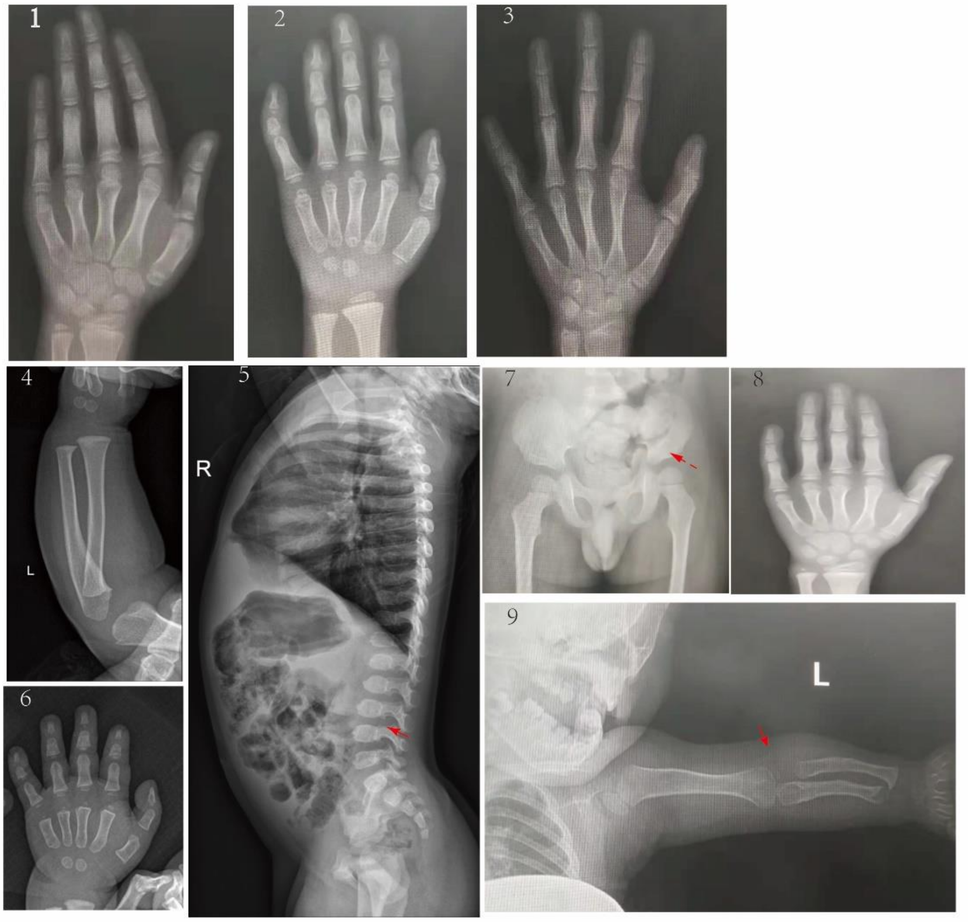

Figure S1 X-ray imaging performance of five patients, **S1-3** showed patient 1-3 left hand X-ray at 13, 7, 5 years old, respectively. **S4-6** showed X-ray of patient 4 imaged at 1 year old, who harbor the compound heterozygous R363* & F857S *NPR2* variants, show that vertebrae are severely compressed and deformed. The bone age is 3 years old. **S7-9** showed X-ray of patient 5 imaged at 2 years old, who has Y306S& R557C in *NPR2*, shows bilateral iliac bones are shortened, acetabular rim is unregular (red arrow), as well as the widening of backbone and metaphysis (red arrow). The bone age of patient 5 is 2 years old.
